# Effect of Acute Sprint Exercise on Myokines and Food Intake Hormones in Young Healthy Men

**DOI:** 10.3390/ijms21228848

**Published:** 2020-11-23

**Authors:** Jan Bilski, Agnieszka Irena Mazur-Bialy, Marcin Surmiak, Magdalena Hubalewska-Mazgaj, Janusz Pokorski, Jacek Nitecki, Ewa Nitecka, Joanna Pokorska, Aneta Targosz, Agata Ptak-Belowska, Jerzy A. Zoladz, Tomasz Brzozowski

**Affiliations:** 1 Department of Biomechanics and Kinesiology, Faculty of Health Sciences, Jagiellonian University Medical College, 20 Grzegorzecka Street, 31-531 Cracow, Poland; jan.bilski@uj.edu.pl (J.B.); agnieszka.mazur@uj.edu.pl (A.I.M.-B.); janusz.pokorski@uj.edu.pl (J.P.); jacek.nitecki@uj.edu.pl (J.N.); ewa.nitecka@uj.edu.pl (E.N.); joanna.pokorska@uj.edu.pl (J.P.); 2 Department of Physiology, Faculty of Medicine, Jagiellonian University Medical College, 16 Grzegorzecka Street, 31-531 Cracow, Poland; marcin.surmiak@uj.edu.pl (M.S.); madzia_hubalewska@wp.pl (M.H.-M.); aneta.targosz@uj.edu.pl (A.T.); agata.ptak-belowska@uj.edu.pl (A.P.-B.); 3 Department of Muscle Physiology, Chair of Physiology and Biochemistry, Faculty of Rehabilitation, University School of Physical Education, 31-571 Cracow, Poland; jerzy.zoladz@awf.krakow.pl

**Keywords:** appetite hormones, sprint-exercise, ghrelin, nesfatin-1, irisin, interleukin-6, pancreatic polypeptide

## Abstract

Physical exercise is known to influence hormonal mediators of appetite, but the effect of short-term maximal intensity exercise on plasma levels of appetite hormones and cytokines has been little studied. We investigated the effect of a 30 s Wingate Test, followed by a postprandial period, on appetite sensations, food intake, and appetite hormones. Twenty-six physically active young males rated their subjective feelings of hunger, prospective food consumption, and fatigue on visual analogue scales at baseline, after exercise was completed, and during the postprandial period. Blood samples were obtained for the measurement of nesfatin-1, ghrelin, leptin, insulin, pancreatic polypeptide (PP), human growth factor (hGH) and cytokine interleukin-6 (IL-6), irisin and plasma lactate concentrations, at 30 min before exercise, immediately (210 s) after exercise, and 30 min following a meal and at corresponding times in control sedentary males without ad libitum meal intake, respectively. Appetite perceptions and food intake were decreased in response to exercise. Plasma levels of irisin, IL-6, lactate, nesfatin-1 and ghrelin was increased after exercise and then it was returned to postprandial/control period in both groups. A significant rise in plasma insulin, hGH and PP levels after exercise was observed while meal intake potentiated this response. In conclusion, an acute short-term fatiguing exercise can transiently suppress hunger sensations and food intake in humans. We postulate that this physiological response involves exercise-induced alterations in plasma hormones and the release of myokines such as irisin and IL-6, and supports the notion of existence of the skeletal muscle–brain–gut axis. Nevertheless, the detailed relationship between acute exercise releasing myokines, appetite sensations and impairment of this axis leading to several diseases should be further examined.

## 1. Introduction

It is generally accepted that body mass depends on a balance between food intake and energy expenditure and that long-term energy surplus leads to obesity, now recognized as the main economic and public health burden [1]. Epidemiological studies use the body mass index (BMI) to assess obesity at the population level. BMI allows for a reliable assessment of health risk at the population level, while at the individual level, apart from total obesity, the distribution of adipose tissue and a number of genetic and socio-economic factors should also be taken into account. The serious bias and stigma faced by obese people can also contribute to their increased morbidity and mortality [2]. The control of energy balance has been attributed mainly to the regulation of the appetite sensations hunger and satiety [3]. The mechanisms underlying feeding behavior are largely unknown; however, the neuroendocrine system, psychological factors and body composition may play an essential role in response to dietary and exercise interventions [3]. 

The hunger and satiety sensations could be influenced not only by food intake but also by physical exercise [4,5,6]. It has been shown that single bouts of exercise can evoke a short-lived inhibition of subjective hunger sensation exerting a relatively small effect on absolute energy intake following exercise [4]. The type of exercise, however, may be an essential factor determining the effects of acute exercise on appetite sensations and energy intake (EI). A meta-analysis addressing the impact of acute exercise on the release of hormones related to appetite regulation concluded that acute exercise caused transient inhibition of the release of acylated ghrelin, the only known orexigenic gut peptide and growth hormone-releasing hormone (GHRH) [5]. An acute bout of exercise concurrently stimulated release of peptide-YY (PYY), glucagon-like-peptide-1 (GLP-1) and pancreatic polypeptide (PP) [6]. However, despite this existing evidence, there is still uncertainty as to how different bouts of exercise can affect appetite regulation, energy intake and the release of GI-tract peptides [4,6]. So far, most studies have focused on moderate-intensity aerobic exercise; however, a clear association between post-exercise hormones release, appetite sensations and energy intake (EI) has not extensively been studied [6]. However, the results of recent studies strongly suggest that that high-intensity exercise leads to so-called “exercise-induced anorexia” with subsequent EI decrease via the release of anorexigenic peptides. While leptin and insulin were proposed to play an essential role in the tonic regulation of appetite, the gut peptides have generally been considered as episodic mediators of hunger and satiety [7]. 

Bouts of high-intensity exercise resulting in profound disturbances of muscle metabolic stability [8,9,10] are commonly performed by athletes who specialize in various sports disciplines, e.g., (sprints running, cycling, soccer, tennis etc.) Moreover, within the last decade, sprint interval training has aroused great interest as an alternative to endurance exercise [11,12]; however, its effect on hormonal and energy intake and hunger sensation has not been fully elucidated, and only a few studies are available addressing the effect of a single bout of sprint exercise on these parameters [13].

Little is known about the role of nesfatin-1, a recently discovered anorexigenic polypeptide derived from posttranslational processing of the nucleobindin 2 (NUCB2) genes in the regulation of appetite and EI [14]. The expression of nesfatin-1 has been detected in the stomach, predominantly in gastric X/A-like cells but in different vesicles than ghrelin. It was suggested that nesfatin-1 could exert the inhibitory effect on the orexigenic action of ghrelin [15]. It is of interest that nesfatin-1 is also expressed in adipose tissue, and its role as adipokine has been postulated [16]. In previous studies, the opposite effects of exercise on plasma nesfatin-1 levels have been observed [17,18,19,20]. Some authors described an increase in plasma nesfatin-1 following exercise [17,18], while others reported no change [19] or even a reduction in plasma nesfatin-1 [20]. These contradictory results can be explained by the different bouts of exercise designed and conducted in these studies [17,18,19,20].

Exercise can influence the appetite and food intake control by affecting the release of skeletal muscle-derived peptides stimulated by these muscle contractions [21,22]. It was suggested that myokines released from working skeletal muscle exert their beneficial metabolic effects through crosstalk between skeletal muscle and different organs, including adipose tissue [21,22,23,24]. The involvement of the interleukin (IL)-6, which during exercise is released predominantly from contracting muscles [22], has been proposed to play a role in the regulation of appetite and energy intake [25,26]. Furthermore, results from animal studies suggest the importance of irisin in the regulation of appetite [27]. This study was designed to evaluate an impact of 30 s all-out maximal intensity cycling on the subjective feelings of hunger sensations, ad libitum food intake and plasma levels of key hormones implicated in post-exercise regulation of appetite and food intake (acylated ghrelin, nesfatin-1, PP, leptin and insulin), potentially muscle-derived peptides (IL-6, irisin) and human growth hormone (hGH). In the present study, as a model of short-term maximal exercise, we have used the standard cycling 30-s all-out Wingate Test [28], which also allowed us to determine the magnitude of power generated by the studied volunteers during the 30 s sprint.

Based on previous studies, we hypothesized that hunger sensations and energy intake would be altered by short term bout of exhaustive exercise and that this would be associated with changes in blood concentration of tested hormones.

## 2. Results

### 2.1. Exercise Performance

The maximal power output reached during any given 5 s period of the test (MPO_5-s_), expressed in watts per kilogram of body mass (W·kg·BM^−1^), are presented in Table 1. The values of the mean power generated throughout the 30 s period of the test (MPO_30-s_) expressed in watts per kilogram body mass (W·kg·BM^−1^) are presented in Table 1. No significant changes in either hemoglobin or hematocrit were observed over time during the exercise or resting sessions (not shown). Thus, the hemoconcentration due to, for instance, the enhanced loss of plasma volume into interstitial space commonly considered as the biomarker in high-intensity exercise [29] was unlikely to occur during the exercise sessions performed in our present study.

### 2.2. Appetite Perceptions

**Figure 1:**
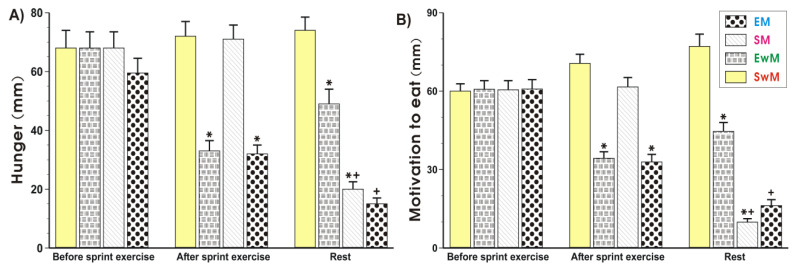
Perceptions of hunger (**A**) and motivation to eat (**B**) in sedentary and exercising human subjects. Values represent means ± SD for 26 subjects. Statistical analysis was done by two-way repeated measures ANOVA and Tukey post hoc test. An asterisk indicates a significant decrease (*p* < 0.05) as compared with baseline values at the initiation of the test and with SwM and SM groups before meal consumption. Asterisk and cross indicate a significant decrease (*p* < 0.05) as compared with respective values in group SwM and group SM before meal consumption. Cross indicates a significant decrease (*p* < 0.05) as compared with respective values in the EM group before meal consumption and the EwM group. EM—the 30 s Wingate Test (W-T30) with ad libitum test meal intake, EwM—W-T30 without ad libitum test meal, SM—sedentary with ad libitum test meal intake, SwM—sedentary without ad libitum test meal intake.

**Figure 2:**
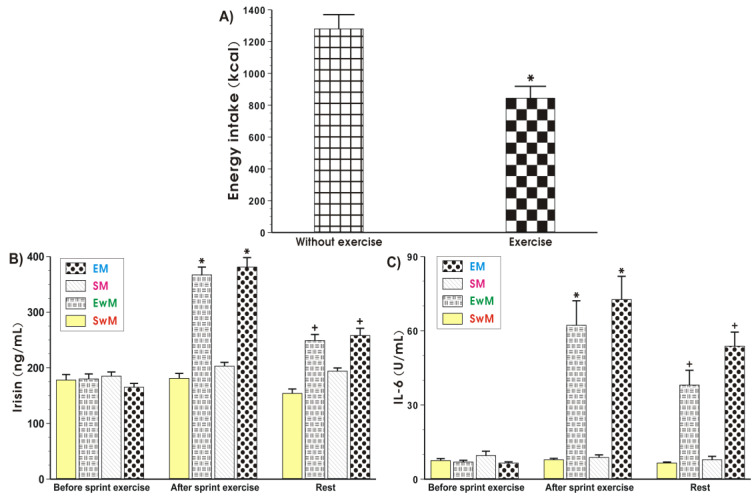
Energy intake at the test meal during exercise and resting sessions (**A**), and the plasma irisin (**B**) and IL-6 (**C**) levels determined at basal sedentary conditions and after the Wingate exercise in subjects with or without meal feeding. Values represent means ± SD for 26 subjects. Statistical analysis was done by paired t-Student test for data presented in Figure 2A. Asterisk indicates a significant decrease (*p* < 0.05) as compared with values obtained in subjects without exercise. For data presented in Figure 2B,C, the statistical analysis was done by two-way repeated measures ANOVA and Tukey post hoc test. An asterisk indicates a significant increase (*p* < 0.05) as compared with basal values. Cross indicates a significant decrease (*p* < 0.05) as compared with respective values in groups EwM and EM before meal consumption. EM—the 30 s Wingate Test (W-T30) with ad libitum test meal intake, EwM—W-T30 without ad libitum test meal intake, SM—sedentary with ad libitum test meal intake, SwM—sedentary without ad libitum test meal intake.

As shown in Figure 1 and Figure 2, the subjective sensations such as hunger (“how hungry do you feel?”) and motivation to eat (“how much do you feel you can eat?”) ratings did not significantly differ in control subjects at rest prior to exercise. The hunger and motivation to eat scores were significantly decreased (*p* < 0.05) immediately upon cessation of the sprint exercise and this effect was maintained at 30 min after exercise (Figure 2A,B). Meal ingestion significantly decreased the hunger sensations and the motivation to eat in non-exercising resting subjects compared with the values obtained in sedentary control subjects without a meal (*p* < 0.05) (Figure 2A,B). Following the test meal, hunger, and motivation to eat ratings significantly decreased as compared with respective values in exercising subjects without a meal (*p* < 0.05) (Figure 2A,B). The motivation to eat was significantly decreased in non-exercising subjects ingesting a test meal as compared with sedentary ones without a meal (*p* < 0.05) (Figure 2A,B).

### 2.3. Energy Intake (EI)

The EI at the test meal was significantly lower after exercise when compared to the sedentary groups without exercise (Figure 2A).

### 2.4. Effect of Exercise on Plasma Hormones Concentrations

As shown in Figure 2B, the baseline values of plasma irisin levels were similar in all tests in basal conditions. In contrast, the plasma irisin concentration was significantly increased in response to exercise and significantly decreased after that (*p* < 0.05). In sedentary trials, the plasma irisin concentration was not significantly altered after meal consumption. The plasma levels of this myokine in these subjects were similar to these recorded in sedentary subjects without a meal (Figure 2B). Meals being given to exercising subjects caused a significant reduction in the plasma level of irisin in exercising and non-exercising subjects comparing with the respective values of this myokine in exercising groups with a meal (EM) and without a meal (EwM) before meal consumption (*p* < 0.05) (Figure 2B).

At basal conditions, before exercise, the plasma IL-6 levels were negligible (Figure 2C). In response to exercise a significant rise in the plasma IL-6 level was observed (*p* < 0.05) (Figure 2C). Meals given to sedentary subjects (SM group) failed to significantly affect the plasma IL-6 levels as compared with sedentary individuals without a meal (SwM group) at each time study point (Figure 2C). After meal ingestion, the plasma IL-6 concentration was significantly decreased in groups EM and EwM comparing with respective values before meal ingestion (*p* < 0.05) (Figure 2C).

**Figure 3:**
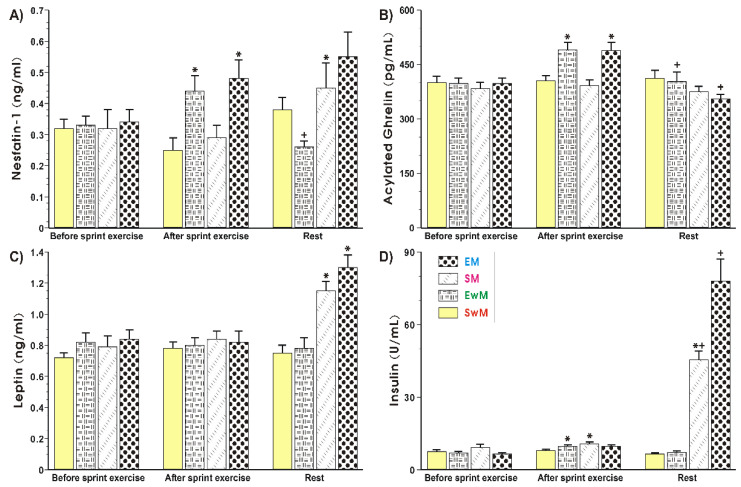
Plasma nesfatin-1 (**A**), acylated ghrelin (**B**), leptin (**C**) and insulin (**D**) levels determined at basal conditions and after exercise in subjects with or without meal consumption. Values represent means ± SD for 26 subjects. Statistical analysis was done by two-way repeated measures ANOVA and Tukey post hoc test. An asterisk indicates a significant change (*p* < 0.05) as compared with the respective values recorded in individuals at the initiation of the test. Asterisk and cross indicate a significant increase (*p* < 0.05) as compared with respective values in group SM before meal consumption and those obtained vs SwM group. Cross indicates a significant change (*p* < 0.05) as compared with respective values in group EwM before and after exercise. EM—the 30 s Wingate Test (W-T30) with ad libitum test meal intake, EwM—W-T30 without ad libitum test meal intake, SM—sedentary with ad libitum test meal intake, SwM—sedentary without ad libitum test meal intake.

Figure 3A shows that plasma nesfatin-1 levels remained unchanged for all resting conditions at the baseline. A significant increase (*p* < 0.05) in plasma nesfatin-1 levels was observed in exercising subjects (EM and EwM) and this effect was maintained after meal consumption in group EM, but plasma nesfatin-1 was significantly decreased after the end of exercise in our group as compared with the values before meal ingestion (*p* < 0.05) (Figure 3A). The plasma nesfastin-1concentration was unaffected with meal ingestion by sedentary subjects without exercise (SwE group) (Figure 3A). In exercising subjects fed with a test meal, the plasma concentration of nesfatin-1 reached similar values to those in the sedentary group of subjects fed a meal (group SM), but the difference between these two groups failed to reach statistical significance (Figure 3A). There was a significant increase in exercising subjects with meal ingestion as compared with respective values of this peptide observed in exercising subject without meal ingestion (*p* < 0.05) (Figure 3A).

As shown in Figure 3B, the plasma levels of acylated ghrelin were not significantly different between all controls recorded at the test initiation. The plasma ghrelin content rose significantly in response to exercise and decreased after that in EwM and EM groups before meal consumption (*p* < 0.05). After meal consumption, a significant decrease in plasma ghrelin level was observed as compared with respective values in exercising subjects (*p* < 0.05) (Figure 3B). The plasma leptin levels remained without statistical differences for all resting conditions at the baseline (Figure 3C). Exercise had no significant effect on plasma leptin levels tested in all groups of subjects. In subjects with a consumed meal, a significant rise in plasma leptin levels was observed as compared with the plasma leptin levels in exercising subjects without meal consumption (*p* < 0.05) (Figure 3C). The plasma insulin levels showed similar values in all control groups recorded at baseline (Figure 3D). The small but significant increase in plasma insulin levels was observed after exercise compared with subjects at resting conditions (*p* < 0.05). In subjects fed with a test meal after exercise, a further significant rise in plasma insulin levels was noticed (*p* < 0.05) (Figure 3D).

**Figure 4:**
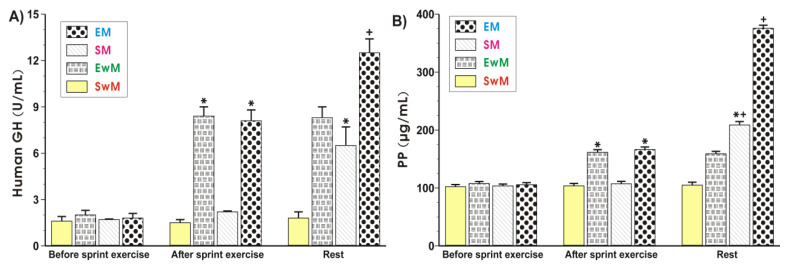
The alterations in plasma human growth hormone (hGH) **(A**) and pancreatic polypeptide (PP) **(B**) levels determined at basal conditions and after the end of exercise in subjects with or without meal consumption. Values represent means ± SD for 26 subjects. Statistical analysis was done by two-way repeated measures ANOVA and Tukey post hoc test. An asterisk indicates a significant increase (*p* < 0.05) as compared with initial values at the beginning of the test. Asterisk and cross indicate a significant increase (*p* < 0.05) as compared with respective values in group SM before meal ingestion. Cross indicates a significant increase (*p* < 0.05) as compared with respective values in group EM before meal consumption. EM—the 30 s Wingate Test (W-T30) with ad libitum test meal intake, EwM—W-T30 without ad libitum test meal intake, SM—sedentary with ad libitum test meal intake, SwM—sedentary without ad libitum test meal intake.

As shown in Figure 4A, the plasma hGH was similar in all control groups at resting conditions. Exercise resulted in a significant increase in the plasma levels of hGH (*p* < 0.05), and this increase also persisted at 30 min after exercise. Following the test meal, the plasma hGH content was further significantly increased in exercising subjects as compared with those who did not exercise (Figure 4A). The results of plasma PP levels in sedentary and exercising subjects with and without a meal are presented in Figure 4B. The plasma PP levels reached similar values in all control groups at rest. In contrast, a significant increase in plasma PP was observed in exercising subjects compared with sedentary ones (*p* < 0.05) (Figure 4B). Subjects fed a meal following exercise presented a significant increase (*p* < 0.05) in plasma level of PP compared with respective values recorded in subjects with exercise but without a test meal (Figure 4B). Subjects who were fed a test meal presented similar plasma concentrations of PP to those recorded in sedentary individuals (Figure 4B).

## 3. Discussion

The purpose of this study was to examine the effects of maximal short-term exercise as reflected by a single 30 s all-out Wingate test bout on pre-prandial and postprandial levels of appetite hormones. These hormones, such as acylated ghrelin, nesfatin-1, PP, leptin and insulin, as well as myokines, such as IL-6, irisin and hGH, were recently implicated in post-exercise control of food intake (32). In particular, we have attempted to provide a potential mechanism for the expected fall in appetite sensations and EI in response to exercise. It should be underlined that the present study involved a group of young, healthy men with high short term power generating capabilities (see Table 1), who could adequately tolerate this very demanding bout of exercise. Taking into consideration that the (MPO_5-s_) of our volunteers, as presented in Table 1, represents the maximal power output reached during any given 5 s period of the Wingate Test, one would expect that their true maximal peak power (MPO) [30] could be a few percent higher than the MPO_5-s_ [31].

To our best knowledge, we have provided for the first time evidence that the acute maximal-intensity and short-duration bout of exercise caused an increase in plasma nesfatin-1 levels under fasting conditions of normal-weight subjects, with a concomitant reduction in self-reported hunger. Interestingly, the 30 s bout of all-out sprint diminished EI with ad libitum test meal intake in these subjects. Another important finding was that an acute bout of exercise increased the plasma levels of PP. Moreover, no significant changes in plasma leptin levels were noticed, but plasma hGH, insulin, IL-6, irisin and lactate concentrations were significantly elevated following the 30 s all-out sprint. We have also observed a slight increase in plasma ghrelin levels in our exercising subjects. It is nowadays clearly established that episodic hormones, mainly gastrointestinal peptides released in response to feeding, or those controlling the anticipation of eating are involved in the mediation of short-term appetite response. The majority of these hormones play an essential role in the mechanism of appetite inhibition, and only ghrelin has been shown to stimulate appetite sensations [7].

The phenomenon of transient reduction in perceived hunger that may be observed for a period of time after intense exercise was initially called “exercise-induced anorexia”; however, this term has also been referred to the time after moderate-intensity exercise [32]. The mechanism of this phenomenon is not fully understood, but inhibition of ghrelin secretion from the stomach, as well as an increase in the secretion of PYY and GLP-1 by the small intestine or PP release from pancreas linked with the decrease splanchnic blood flow, were proposed to explain this exercise-induced anorexia [33]. Furthermore, the release of PYY, GLP-1 and PP could affect the desire to eat by a decrease in the gastric emptying rate [7].

Despite this fact that the effect of acute exercise on energy intake has been studied extensively, data are relatively equivocal, and changes in hunger sensation in exercising individuals do not always correlate with changes in EI after exercise [4]. Herein, we have observed that the short, acute exercise caused a marked reduction in both EI and hunger sensations. Moreover, these effects were associated with an inhibition of appetite and the decreased motivation to eat noticed in our subjects right after the end of a single bout of exercise. Among appetite hormones, ghrelin has been reported as the only known orexigenic gut peptide which has been proposed as a significant factor responsible for this “exercise-induced anorexia”. Although the acylated form of the peptide represents only 10% of circulating ghrelin, this form is believed to be responsible for ghrelin orexigenic actions [5]. Ghrelin can act *via* growth hormone secretagogue receptors (GHS-R) also known as ghrelin receptors, located on the vagal nerve [34] and hypothalamic nuclei [35] to stimulate food intake. The relationship between acute exercise and total ghrelin plasma appears inconclusive since the diminished release of acylated ghrelin was initially reported [6]. However, depending on the type of exercise, the duration of exercise tests and subjects participating in these studies, no changes or even small increase in the plasma acylated ghrelin levels has been documented [36,37,38]. In agreement with some of these data, we have noticed a small but significant increase in the acylated ghrelin plasma level after a single all-out 30 s bout of exercise, but the plasma ghrelin level returned to the basal values at post-exercise resting conditions. Indeed, Broom et al. [39] have confirmed that the acylated ghrelin response to acute exercise depends on exercise intensity and duration. We assume that the short-duration of exercise in our present study could account for the lack of inhibitory effect of physical exercise on the release of ghrelin. Utilizing the mouse model of treadmill running, Mani et al. [40] have observed that a short bout of high-intensity exercise produced a transient increase in the plasma of acylated ghrelin.

On the other hand, one of the proposed hypotheses of the post-exercise decrease in acylated ghrelin release could be a noticeable reduction in splanchnic blood flow which is commonly associated with acute exercise [41]. We found that ghrelin is increased in exercising subjects, and it had decreased thereafter. The selected duration of exercise in our study design might be too short to evoke more pronounced inhibition in plasma ghrelin. Inhibitory action of insulin on ghrelin release was postulated as another possible mechanism of post-exercise fall in plasma ghrelin level [42]. The infusion of insulin has been shown to rapidly suppress circulating ghrelin concentrations while sprint exercise caused an increase in plasma insulin levels; this phenomenon was also observed in our present study. We propose that the acylated ghrelin could also be responsible for the stimulation of hGH release. In this regard, ghrelin appears to be an even more potent stimulus for growth hormone release than growth hormone-releasing hormone (GHRH) [43]. In our present study, short, high-intensity exercise has evoked a marked increase in circulating hGH, and this observation is corroborative with existing evidence [44]. However, to our knowledge, this is the first study that has shown that meal ingestion after exercise can further enhance the stimulatory effect of exercise on the release of hGH.

The evident decrease in appetite sensations and EI in response to exercise can be attributed to the increased plasma concentration of PP, the GI-hormone which released under vagal control and secreted in a biphasic fashion in proportion to food intake. The inhibitory effect of PP on food intake in humans seems to be indirectly regulated through afferent vagal activity, in part, by decreasing gastric emptying [45]. Only a single study has examined the PP response to different bouts of exercise and documented a substantial rise in PP plasma level following exercise [6]. Herein, we have documented the PP response to sprint exercise in subjects under fasting condition and later fed with a meal. We have found that sprint exercise markedly increased PP release in fasting individuals and this pre-prandial exercise potently augmented the plasma PP response to a meal.

As mentioned before, the novel satiety hormone nesfatin-1 could be in part responsible for anorexigenic action of exercise. Nesfatin-1 has been initially discovered in the hypothalamus, but the immunoreactivity of nesfatin-1 has also been identified in peripheral tissues, including the stomach. Nesfatin-1 was reported to decrease food intake upon the injection of this peptide into the third ventricle but also following its peripheral administration [46]. Peripheral nesfatin-1 can enter the brain appetite centres from the circulation after crossing of the blood-brain barrier, or it can modulate the vagal afferent activity targeting brain structures via the gut–brain axis [46]. Our data demonstrated that plasma nesfatin-1 concentrations were significantly elevated following acute exercise. Therefore, we suggest that this effect can be partly responsible for the exercise-induced decrease in appetite sensation, including the inhibitory effect on both the hunger and the motivation to eat.

It has been demonstrated that IL-6 is released from skeletal muscle in a contraction-dependent manner [21] and that IL-6 is involved in an inhibitory effect of exercise on appetite sensation and food intake [25]. IL-6 has also been proposed to play a role in the mechanism of the stimulation of GLP-1 and PYY release [47]. Furthermore, IL-6 can affect hunger sensation and food intake, acting centrally at the level of the hypothalamus [48]. IL-6 could stimulate the release of other appetite-regulating peptides, and a role of IL-6 as a mediator of the effects of exercise intensity on hunger suppression has recently been suggested [49]. Since in our study, a marked increase IL-6 plasma level has been observed, it is quite possible that this cytokine is responsible, at least in part, for the observed post-exercise reduction in EI and appetited sensations. Although contracting muscles contribute to most of the IL-6 present in the circulation in response to exercise, the other sources of IL-6, including leucocytes, have been proposed to contribute to the blood release of this cytokine [47].

Exercise is known to upregulate the expression of peroxisome proliferator-activated receptor-γ coactivator 1α (PGC-1α), which subsequently triggers a proteolytic cleavage of FNDC5 and release of irisin from skeletal muscle into the circulation in experimental animals and human subjects [50]. Irisin derived either from muscle or from the brain can mediate the complex bodily response to physical exercise. In our opinion, such a mechanism might operate instead in the time-course of physical training but not after a single bout of short term exercise, since it seems to be rather unlikely that the single bout of 30 s sprint exercise could so rapidly increase the level of PGC-1α protein in skeletal muscle [12], which is needed to initialize this process. It is of interest that intra-hypothalamic injection of irisin resulted in inhibition of food intake and this effect was associated with a significant reduction in orexin-A and an increase in expression of pro-opiomelanocortin (POMC) gene in the hypothalamus [50]. In our study, the extensive irisin release in sprint exercising individuals has been observed, supporting the notion that irisin plays a vital role in the control of appetite and food intake in post-exercising human subjects.

Recently, Islam et al. [26] have underlined the role of lactate in the mediation of “exercise-induced anorexia”. It is already well known that administration of exogenous lactate has an anorexigenic effect in humans and animals [51], which can be, at least in part, mediated by lactate binding to ghrelin-producing cells, thus inhibiting ghrelin release [26,49]. The question remains as to whether hormone release by fit (normal fit not professional athletes) and unfit individuals in our present study would yield similar results. The evidence based medicine shows mixed results, in part because there are many confounding factors, such as body composition (i.e., high content and distribution of fat), different development of muscle tissue, yet of course, sex and age. Most of the results indicate that under baseline conditions, there are no significant differences between unfit vs. normal fit, but they may significantly differ in response to exercise. One such example is the plasma level of hGH. Sutton et al. have observed that there was an elevation of hGH level during exercise in both fit and unfit participants, but in the fit group hGH returned to basal levels within 30 min, whereas in the unfit group the hGH level continued to rise for next hours [52]. A recent meta-analysis has shown that being fit is associated with a nearly two fold increase in post-exercise irisin concentration, compared with individuals considered as unfit [53].

It should be underlined that the present study involved young, healthy men, with relatively high exercise capacity as judged by the power output generated during a 30-s all-out sprint; this is why the conclusions of the present cannot be simply extrapolated to all human subjects. Furthermore, the mode of physical exercise applied in our study should not be recommended to everybody, since due to its stressful nature and very high metabolic demands it could be harmful to the health status of several groups of people, especially to patients and the older individuals.

## 4. Materials and Methods

### 4.1. Study Participants

Twenty six moderately active, non-smoking male subjects, firefighters trainees volunteered to participate in this study. They were billeted at firefighters’ training center and had precisely the same diet and daily schedules, including moderate, daily physical activity. None of the subjects had signs or symptoms of acute or chronic disease or was taking any medications, and none were undertaking regular sprint-type training on a daily basis. The subjects’ age was 28.7 ± 4.1, and their height and body mass (BM) were 1.78 ± 0.1 m and 87.2 ± 11.7 kg, respectively. The recruited subject’s body mass index (BMI) reached a value of 27.1 ± 2.6 kg/m^2^, indicating that these study participants were slightly overweight. The volunteers were fully informed of the study design and gave their informed written consent before participation. The study protocol was approved by the Jagiellonian University Bioethics Committee (KBET/228/B/2012 issued on 27 June 2012 and extended on 9 May 2013) and conformed to the guidelines set forth by the Declaration of Helsinki.

### 4.2. Study Design and Experimental Protocol

**Figure 5:**
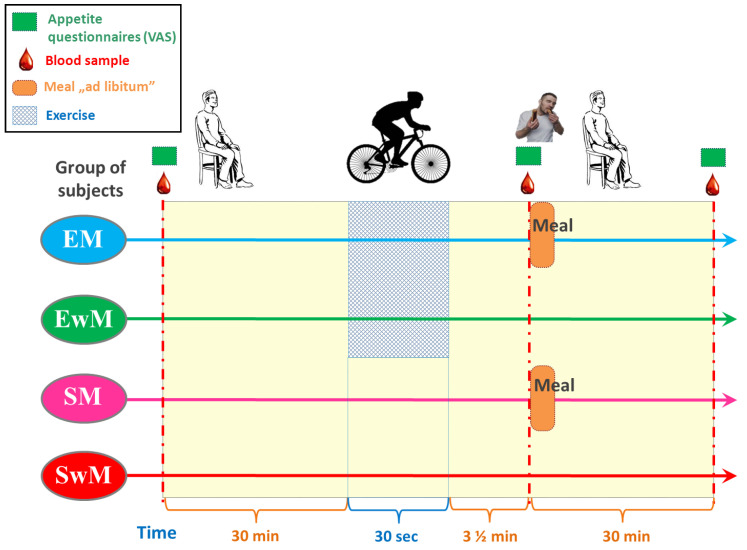
Schematic flowchart of the study design.

A within-subject, crossover clinical trial design was utilized for the study based on the recommendation for such studies [51] and participants completed all four sessions in a randomized order. Experimental sessions consisted of two exercise sessions: (1) the 30 s Wingate Test with ad libitum test meal intake, (2) W-T30 without ad libitum test meal intake, and two sedentary sessions (participants rested for the entire duration of the trial): (3) with ad libitum test meal intake, (4) without ad libitum test meal intake (Figure 5). EwM and SwM trials were used as control tests for respective groups with exercise in exercising and sedentary subjects.

Sessions were conducted between 9 a.m. and 10 a.m. after a 12-h overnight fast and participants were allowed at least two weeks of washout between two different sessions.

The tests were preceded by a familiarization session in which participants completed demographic and health history questionnaires and underwent anthropometric and health testing. In addition, participants were familiarized with the Wingate test and VAS scales during ad libitum test meal intake.

Subjects were requested to consume the same, standard isocaloric meal at least 12 h before each test session. Only water was allowed ad libitum. They were requested to maintain their habitual physical activity. They were also asked to avoid strenuous activity and to avoid alcohol and caffeine during the 24 h preceding each test. The compliance with these directions was easy to control because all the participants were billeted at the firefighters’ training center and had exactly the same diet and daily schedules. Throughout the testing period, the mean ambient temperature and relative humidity of the laboratory were kept stable (21.7 ± 1.1 °C and 48.2 ± 4.1%, respectively).

### 4.3. Exercise with the W-T30

W-T30-s was conducted on a friction-loaded cycle ergometer (Monark Exercise AB 894E Peak Bike Anaerobic Testing Ergometer, Varberg, Sweden) equipped with Windows-based software and interfaced with a computer. The seat height and handlebars were adjusted appropriately for each subject. The W-T30-s consisted of a 30 s maximal sprint against a constant resistance related to body mass (7.5% of body mass). On the day of the test, the W-T30-s began from a rolling start, at 60 rpm against minimal resistance (weight basket supported). When a constant pedal rate of 60 rpm was achieved, a countdown was started. With less than 1 s left in the countdown, test resistance was instantaneously applied. All subjects were verbally encouraged to continue to pedal as fast as they could for the entire 30 s. Every second, power output was calculated by the computer and stored. The highest power output over 1 s corresponding to the ratio between total work done and time taken to do it (i.e., 30 s), was recorded at the end of the test.

Either exercise or sedentary study was followed by the test meal and by a postprandial observation or in control test, during the resting time. During the resting condition, participants remained seated, and were allowed to read/write quietly.

### 4.4. Visual Analog Scales and Food Intake

Visual Analog Scales (VAS) were employed to assess subjective appetite sensations. Based on current recommendations [54] we have decided to test “Hunger” and “Motivation to Eat”. Subjects rated their perceived feelings of hunger and motivation to eat on paper-based 100 mm visual analogue scales (VAS) anchored at each end with contrasting statements, at baseline, exercise, and during the postprandial period, respectively, as described previously [55]. These VAS scales were found to be valid and reproducible for assessing appetite perceptions [55].

Based on the available literature, we decided to use a single course and not the buffet style as it is generally recommended to assess short-term energy compensation [54]. Participants were provided with ad libitum meal, consisting of sandwiches consisting of bread, butter and ham (2.73 kcal.g-1, energy percentages: 44.4% carbohydrate, 16.2% protein and 39.4% fat) which they were instructed to eat until reaching satiety. All food presented was pre-weighed, and upon the completion of the meal, all food was re-weighed to the nearest 0.1 g and energy content (kcal) was determined in next 15 min, as described previously [55].

### 4.5. Borg’s Rating of Perceived Exertion Scale (6–20)

Borg’s Rating of Perceived Exertion (RPE) Scale 6–20 scale is the most widely used instrument to measure perceived exertion or exercise intensity and was used in our study to assess the subjective perception of effort directly after the end of each bout of exercise [56].

### 4.6. Blood Collection and Biochemical Measurements

In all tests, the venous blood samples were obtained from an antecubital vein, at 30 min before exercise, immediately (210 s) after exercise, and 30 min following a meal (or at corresponding times in control tests) (Figure 5). The blood samples were collected in the test tubes containing ethylenediaminetetraacetic acid (EDTA) and aprotinin and in tubes without anticoagulants. Test tubes were delivered immediately to the laboratory and centrifuged for 15 min at 3500 g. The plasma was stored at −80°C for further analysis.

The plasma level of hormones was measured using commercially available ELISA kits for cytokine IL-6, leptin and nesfatin-1 (R&D System, Oakville, Canada). The IL-6 detection limit was 3.1 pg/mL and the intra- and inter-assay coefficients of variation for this cytokine were 2.6% and 3.3%, respectively. The leptin detection limit was 15.6 pg/mL and intra- and inter-assay coefficients of variation were 3.3% and 4.2%, respectively, and the nesfatin-1 detection limit 0.78 ng/mL and the intra- and inter-assay coefficients of variation for this hormone were 11.8% and 13.6%, respectively. Acylated ghrelin levels were measured using commercially available ELISA kit (LifeSpan Biosciences, Seattle WA, USA). The intra- and inter-assay coefficients of variation for ghrelin were lower than 6.61% and 5.4%, respectively, and sensitivity was 9.38 pg/mL.

Irisin concentration was measured using a commercial ELISA kit (Phoenix Pharmaceuticals Inc., Burlingame, CA, USA). The intra- and inter-assay coefficients of variation and sensitivity were 5.61% and 14.56% and 0.78 ng/mL, respectively. Plasma levels of insulin and hGH were determined with the use of IRMA assay (Diasource, Nivelles, Belgium and Cisbio, Cedex, France, respectively). The intra- and inter-assay coefficients of variation were for insulin 2.1% and 6.1% and sensitivity 1 µIU/mL; for hGH 2.1% and 5.0%, respectively, and the sensitivity was 0.03 µIU / mL. PP was assessed by radioimmunoassay (RIA) kit (Euro-Diagnostica AB, Sweden). Intra and inter-assay CV were 2.6% and 3.5%, respectively. All tests were performed according to manufacturer recommendations. Hemoglobin and hematocrit were measured at Cracow University Hospital Diagnostic Laboratory using HemoCue Hb201+ (Angelholm, Sweden) and Hawksley Haematospin 1400 (Hawksley & Sons, Ltd., Lancing, UK).

A droplet of blood was taken from one of the tubes and placed on a lactate strip for the measurement of blood lactate using a handheld, battery-powered reflectance photometer (Accutrend lactate meter, Roche Diagnostics, Mannheim, Germany).

### 4.7. Statistical Analysis

All calculations were made using Statistica software v. 10 (StatSoft 2011, Tulusa, OK, United States. The normality of the data distribution was verified by means of Kolmogorov–Smirnov test, and results are expressed as means ± SD. Statistical analysis was done using two-way repeated measures ANOVA with Tukey post-hoc test to study the differences between the study conditions (rest, before and after sprint exercise) and the differences between the study groups. In addition, the paired t-test was used to compare the difference in energy intake between exercise and without exercise conditions. Differences of *p* < 0.05 were considered significant.

## 5. Conclusions

The present study showed that acute short-term fatiguing exercise could transiently suppress hunger sensations and food intake in humans. This adaptive response seems to be related to exercise-induced alterations in plasma hormones and myokines, as presented in our study. Our finding suggests that a short-term maximal exercise should be considered as a potential physiological intervention for the treatment of disturbances in the mechanism of food control intake in humans. Future research is warranted to study these responses in obese individuals for whom physical exercise is recommended as a complementary therapeutic strategy for weight control.

The present study showed that an acute short-term fatiguing exercise can transiently suppress hunger sensations and food intake in humans. We postulate that this physiological response involves exercise-induced alterations in plasma hormones and myokines such as irisin and IL-6 released, and supports the notion of existence of the skeletal muscle–brain–gut axis. Nevertheless, the detailed relationship between acute exercise releasing myokines, appetite sensations and inactivity leading to serval diseases should be further examined.

## Figures and Tables

**Table 1:** The subjects selected parameters under rest and/or exercise conditions.

Condition	MPO_5-s_ (W·kg·BM^−1^)	MPO_30-s_ (W·kg·BM^−1^)	Plasma Lactate (mmol·L^−1^)	HR (bt·min^−1^)	RPE (6–20)
Rest	–	–	1.07 ± 0.04	71.9 ± 9.1	–
Exercise	10.84 ± 0.95	8.1 ± 0.67	12.4 ± 0.09 *	179.5 ± 15.8 *	17 ± 1.03

Where: MPO_5-s_ is the maximal power output reached during any given 5 s period of the Wingate Test, MPO_30-s_ is the mean power generated throughout the 30 s period of the test. The magnitude of the power outputs is expressed in watts per kilogram of body mass (W·kg·BM^−1^). HR is the heart rate (bt·min^−1^) and the RPE is the Borg’s Rating of Perceived Exertion (scale 6–20). Values are mean ± SD. * indicates a significant difference as compared to the values at resting conditions (*p* < 0.01).
